# Electroacupuncture Could Regulate the NF-**κ**B Signaling Pathway to Ameliorate the Inflammatory Injury in Focal Cerebral Ischemia/Reperfusion Model Rats

**DOI:** 10.1155/2013/924541

**Published:** 2013-07-18

**Authors:** Wen-yi Qin, Yong Luo, Ling Chen, Tao Tao, Yang Li, Yan-li Cai, Ya-hui Li

**Affiliations:** ^1^Department of Neurology, The First Affiliated Hospital of Chongqing Medical University, Chongqing 400016, China; ^2^Chongqing Key Laboratory of Neurology, 1 Youyi Road, Yuzhong District, Chongqing 400016, China; ^3^Department of Neurology, Affiliated Hospital of Luzhou Medical College, Luzhou, Sichuan Province 64600, China; ^4^Department of Oncology, The First Affiliated Hospital of Chongqing Medical University, Chongqing 400016, China

## Abstract

The activated nuclear factor-KappaB signaling pathway plays a critical role in inducing inflammatory injury. It has been reported that electroacupuncture could be an effective anti-inflammatory treatment. We aimed to explore the complex mechanism by which EA inhibits the activation of the NF-**κ**B signal pathway and ameliorate inflammatory injury in the short term; the effects of NEMO Binding Domain peptide for this purpose were compared. Focal cerebral I/R was induced by middle cerebral artery occlusion for 2 hrs. Total 380 male Sprague-Dawley rats are in the study. The neurobehavioral scores, infarction volumes, and the levels of IL-1**β** and IL-13 were detected. NF-**κ**B p65, I**κ**B**α**, IKK**α**, and IKK**β** were analyzed and the ability of NF-**κ**B binding DNA was investigated. The EA treatment and the NBD peptide treatment both reduced infarct size, improved neurological scores, and regulated the levels of IL-1**β** and IL-13. The treatment reduced the expression of IKK**α** and IKK**β** and altered the expression of NF-**κ**B p65 and I**κ**B**α** in the cytoplasm and nucleus; the activity of NF-**κ**B was effectively reduced. We conclude that EA treatment might interfere with the process of NF-**κ**B nuclear translocation. And it also could suppress the activity of NF-**κ**B signaling pathway to ameliorate the inflammatory injury after focal cerebral ischemia/reperfusion.

## 1. Introduction

The pathological mechanism of focal cerebral ischemia/reperfusion is very complex and involves a myriad of distinct molecular signaling and cytokines pathways [1]. As an important and crucial pathological process following focal cerebral ischemia/reperfusion [2, 3], the excessive activation of inflammation could induce the more serious damage, even the fatal injury than ischemic injury itself [4]. Inflammation is a pathological process that occurs via an amplification cascade, the NF-*κ*B signaling pathway [5], and the complex cytokine network is critical for the occurrence of inflammatory responses [6, 7]. Furthermore, more sophisticated approaches are required for the treatment of focal cerebral ischemia/reperfusion because of the narrow therapeutic time window and complex pathological features of this disease [8]. In Asia, especially in China, electroacupuncture (EA) is a general method for the treatment of cerebrovascular diseases. Many reports of basic research and clinical studies have discussed the efficacy of EA in regulating inflammatory injury [9–12]. However, the exact mechanism and functional targets of EA treatment need to be elucidated. Significantly, the mechanism by which EA suppresses the activity of NF-*κ*B signaling pathway at an early stage of cerebral ischemia/reperfusion needs to be discussed.

The NF-*κ*B family of transcription factors consists of five members, p50, p52, p65 (Rel A), c-Rel, and Rel B. NF-*κ*B prototype is a heterodimer composed of the RelA (p65) and NF-*κ*B1 (p50) subunits. This variant is the most potent gene transactivator among the NF-*κ*B family and is the major NF-*κ*B protein found in the nucleus [13, 14]. The inhibitory protein I*κ*B*α*, which masks the nuclear localization signals (NLS) of NF-*κ*B p65 protein and keeps them sequestered in an inactive state in the cytoplasm, leads to constant shuttling of NF-*κ*B/I*κ*B*α* complexes between the nucleus and the cytoplasm [15, 16]. Degradation of I*κ*Bs (especially I*κ*B*α*) is a rapidly induced signaling event that is initiated upon specific phosphorylation of the molecules by activated IKK [16, 17]. The IKK complex mainly contains IKK*α*, IKK*β*, and a regulatory subunit NEMO (NF-*κ*B essential modulator/IKK*γ*). IKK*γ* is not functional in the absence of its interactions with IKK*α* and IKK*β* [18]. Thus, NF-*κ*B p65/I*κ*B*α*, IKK*α*, and IKK*β* are considered to be some of the most important factors involved in activation of the NF-*κ*B signaling pathway during the early stages of focal cerebral I/R. Consequently, we will observe the change of those important factors in order to explore the mechanism of EA treatment for the inflammatory injury after focal cerebral I/R.

The NF-*κ*B specific inhibitor (wild-type) NBD peptide that disrupts the interaction of NEMO (IKK*γ*) with the IKK*β* has some advantages including the following: (1) does not affect basal NF-*κ*B activity, (2) has well-defined molecular sites of action, (3) is specific for the classical NF-*κ*B activation pathway and does not affect the NF-*κ*B alternative pathway, and (4) does not target the active domain of the kinase and is therefore unlikely to affect other essential kinases [19]. The Pharmacology characteristic of NBD peptide prompts a clear understanding about the mechanism of NBD peptide to inhibit excessive NF-*κ*B activation [20]. On the contrary, the mechanism by which EA inhibits the NF-*κ*B signaling pathway activation is not clear. Therefore, we used NBD peptide as a positive intervention method control for IKKs function research in order to investigate the EA mechanism for inhibition of excessive activation of NF-*κ*B after focal cerebral ischemia/reperfusion. Furthermore, the effects of EA and NBD peptide treatment in this process were compared in order to further elucidate the underlying mechanism and targets of EA treatment.

According to the traditional theory of Traditional Chinese Medicine and the basic acupuncture research principle, the “Baihui” (GV 20) and “Siguan” acupoints were chose in this study. The “Siguan” acupoint is composed of the two “Hegu” (LI 4) and two “Taichong” (LR 3) acupoints. Our aim was to define the effective targets and mechanism of EA treatment that influenced the NF-*κ*B signaling pathway and its function in alleviating inflammatory injury after focal cerebral ischemia/reperfusion during the early stages.

## 2. Materials and Methods

### 2.1. Animals

The experimental protocol used in our study was approved by the Ethics Committee for Animal Experimentation of the First Affiliated Hospital of Chongqing Medical University and all procedures were conducted in accordance with the National Institutes of Health Guidelines for Animal Research (Guide for the Care and Use of Laboratory Animals). Male SD rats (280–300 g) were provided by the Experimental Animal Center of the Chongqing Medical University and housed under controlled conditions with a 12-hour light/dark cycle, a temperature at 22 ± 2°C, and humidity at 60% to 70% for at least one week before operation and treatment. The animals were allowed free access to standard rodent diet and tap water. Total 380 rats were randomly divided to four groups: a sham group (*n* = 95), an I/R group (MCAO for 2 h, *n* = 95), an EA group (*n* = 95), and the NBD group (*n* = 95). These groups were assessed from 6 h to 48 h after reperfusion.

### 2.2. Induction of Focal Cerebral Ischemia/Reperfusion

Transient focal cerebral ischemia was induced by MCAO in rats as previously described [21, 22]. Briefly, SD rats were fasted for 12 h but were allowed free access to water before surgery. Anesthetization was induced with 3.5% chloral hydrate (1 mL/100 g) by intraperitoneal injection. The right common carotid artery and the right external carotid artery were exposed through a ventral midline neck incision and were ligated proximally and temporarily. A 2.0 monofilament nylon suture (Ethicon Nylon Suture; Ethicon Inc., Osaka, Japan) with its tip rounded by heating in a flame was inserted through an arteriectomy in the common carotid artery just below the carotid bifurcation and then advanced into the internal carotid artery approximately 18 mm to 20 mm distal to the carotid bifurcation until a mild resistance indicated occlusion of the origin of the anterior cerebral artery and the middle cerebral artery. Reperfusion was accomplished by withdrawing the suture after 2 h of ischemia. The incision was sutured and sterilized. The animals were maintained on the 37°C thermostatic table. The sham surgery was conducted in the ischemia/reperfusion (I/R) surgery groups by insertion of the nylon monofilament suture to 10 mm into the internal carotid artery; do not insert into the middle cerebral artery.

Body temperature was monitored in all animals. The rectal temperature was maintained at 38°C ± 0.5°C throughout all portions of the experiment by means of a rectal thermistor probe and a thermostatically regulated heating lamp placed above the body of the animal. Intrastriatal temperature was monitored and was adjusted by manipulating the height of a small high-intensity lamp placed above the head. Prior to the ischemic insult, intrastriatal temperature was held at 36.5°C ± 0.5°C in all groups during ischemia [23, 24].

### 2.3. Electroacupuncture Treatment

In the EA groups, the total SD rats (*n* = 95) were anesthetized with 3.5% chloral hydrate (1 mL/100 g) by intraperitoneal injection. According to the Experimental Animals Meridians Mapping, the “Baihui” (GV 20)/“Siguan” acupoints were selected, which were located at the intersection of the sagittal midline and the line linking the ears, the second metacarpal midpoint of radial side, and the second toe tibial collateral in the rear of phalanx. These acupoints were stimulated at an intensity of 1 mA and frequency of 2/20 Hz for 30 min using the G6805-2 EA Instrument (Model no. 227033; Beijing Xinsheng Ltd., China). The SD rats were maintained on the 37°C thermostatic table. The EA treatment was given at once each day (from 6 h to 48 h after reperfusion). After ischemia for 2 h, began the first EA treatment when the nylon monofilament was withdrawn. From 6 h to 48 h, the EA treatment is twice, 3 times, 4 times, and 5 times orderly.

### 2.4. Drug Intervention Methods

NBD peptide (ENZO Life Sciences Inc., no. BML-P607-0500, soluble in 50 *μ*L DMSO, final concentration 10 *μ*g/*μ*L) was first time injected intracerebroventricularly 2 h before surgery as previously described [25]. Briefly, the total 72 rats were anesthetized and attached to a stereotaxic instrument (Stoelting, USA). According to stereotactic mapping combined with rat size, we determined the right lateral ventricle position and drilled a cranial window (cross-stitch centered, right shift 1.3 mm, backward 1.5 mm, downward 3.8 mm) for injection of 5 *μ*L NBD peptide once each day, until 48 h after reperfusion. The NBD peptide injection times from 6 h to 48 h are twice, 3 times, 4 times, and 5 times.

### 2.5. Neurobehavioral Evaluation

Neurobehavioral evaluation was carried out in the I/R group, EA groups, and NBD peptide group. After focal cerebral ischemia/reperfusion at different time points, the neurologic deficit scores of model SD rats were assessed by an investigator who was unaware of animal grouping. A modified neurologic deficit score described by Longa et al. was used for neurologic assessment. 0: no deficit; 1: failure to extend left forepaw fully; 2: circling to the left; 3: falling to the left; 4: no spontaneous walking with a depressed level of consciousness. Animals scoring 2 to 3 were included in experiments. Neurobehavioral scores were determined according to the methods described by Garcia et al. [26] and Bederson et al. [27].

### 2.6. Infarct Volume Assessment

The animals (*n* = 5 for each group) were decapitated and 2 mm thick coronal sections from throughout the brain were stained with 2% 2,3,5-triphenyltetrazolium chloride (Sigma-Aldrich) to evaluate the infarct volume, as described by Wang et al. [28].

### 2.7. Immunohistochemistry Analysis

Immunohistochemistry was performed using the avidin-biotin-peroxidase complex method. Briefly, brains were fixed in 4% phosphate-buffered paraformaldehyde (PFA) and immersed into 20% sucrose for 3 h. A 4 mm thick coronal brain slice was cut, beginning 8 mm away from the anterior tip of the frontal lobe. Then a longitudinal cut (from top to bottom) was made approximately 2 mm from the midline through the ischemic hemisphere to remove medial parts. Paraffin-embedded tissues were sectioned and dewaxed for antigens retrieval by immersing slides in 0.01 mol/L citrate buffer, pH 6.0, followed by heating in a microwave oven (temperature 92–98°C) for 20 min. Sections were cooled to room temperature and washed with PBS for 5 min and endogenous peroxides were blocked in 3% H_2_O_2_ for 15 min. Sections were then incubated for 30 min with 5% normal goat blood serum to block nonspecific binding. The primary antibody, NF-*κ*B p65 (C22B4) rabbit mAb (Cell Signaling Technology, 1 : 50), was added and sections incubated at 4°C overnight. The immunostain SP Kit was used. For negative sham group, the primary antibodies were replaced with PBS. A slide image of ischemic penumbra area from each section was scanned and acquired using an OLYMPUS PM20 automatic microscope (Olympus, Tokyo, Japan) and a TCFY-2050 (Yuancheng Inc., Beijing, China) pathology system. The visual fields in each section image (five sections in each brain) were analyzed using the Motic Med 6.0 CMIAS pathology image analysis system (Beihang Motic Inc., Beijing, China).

### 2.8. Double-Immunofluorescence Labeling

Brain tissue was fixed and frozen, and the 10 *μ*m thick coronal brain slices were cut beginning 8 mm from the anterior tip of the frontal lobe. Frozen sections were air dried at 50°C for 30 min. Sections were treated with ten mol/L sodium citrate buffer (pH 6.0) and heated in a microwave oven for 20 min at 92–98°C. Tissues were permeated with 1% Triton X-100, and sections were incubated in 5% goat serum for 1 h at 37°C. Incubations with primary antibodies were carried out at 4°C overnight. The primary antibodies were NF-*κ*B p65 (C22B4) rabbit mAb (Cell Signaling Technology, 1 : 50), I*κ*B*α* (L35A5) mouse mAb (Cell Signaling Technology, 1 : 50), IKK*α* (M-110) (Santa Cruz, no. sc-7183, 1 : 25), and IKK*β* (P-20) (Santa Cruz, no. sc-34673, 1 : 25). After washing, slides were incubated with fluorescent isothiocyanate-conjugated anti-rabbit (1 : 50), anti-mouse (1 : 50), and anti-goat (1 : 100) antibodies for 2 h at 37°C. Finally, sections of ischemic penumbra area were examined by laser-scanning confocal microscopy on an Olympus IX70 inverted microscope (Olympus, Tokyo, Japan) equipped with Fluoview FVX confocal scan head (Leica Microsystems Heidelberg GmbH, Wetzlar, Germany).

### 2.9. Fluorescent Quantitative PCR

Total cellular RNA was isolated with Trizol (Invitrogen, Paisley, UK). cDNA was synthesized with Superscript Reverse Transcriptase (Invitrogen). The polymerase chain reaction was performed with the iQ5 Gradient Real-Time PCR Detection System (Bio-Rad) using primers (Takarad) for NF-*κ*B p65, I*κ*B*α*, IKK*α*, and IKK*β* (Table 1). Data were individually normalized to the mean of the relative expression of GAPDH. Quantitative PCR (Q-PCR) was performed using the Hot Start Fluorescent PCR Core Reagent Kits (SYBR Green I). Each reaction mixture consisted of first-strand cDNA template, 1.25 *μ*L of primer-probe, and 12.5 *μ*L of SYBR Green in a total volume of 25 *μ*L. The following cycling conditions were used: 95°C for 10 min, followed by 40 cycles of 95°C for 5 s and 60°C for 30 s. Subsequently, each sample underwent dissociation curve analysis to examine primer-target specificity as previously described [29].

### 2.10. Western Blot Analysis

Rats were anesthetized with 3.5% chloral hydrate (1 mL/100 g) by intraperitoneal injection. The cortex of the ischemic area was homogenized in RIPA lysis buffer (Beyotime, no. P0013B). Total protein and cytoplasmic/nuclear proteins were extracted on ice using Nuclear and Cytoplasmic Protein Extraction Kits (Beyotime, no. P0027). Western blot analysis was performed with 45 *μ*g protein extract separated by 10% SDS-PAGE, which were then transferred to a polyvinylidene fluoride (PVDF) membrane (Millipore Corporation). Nonspecific epitopes were blocked with 5% skim milk/Tween-20-Tris-buffered saline. The following primary antibodies which were used in double-immunofluorescence labeling analysis: appropriate horseradish peroxidase conjugated antibodies (anti-rabbit (1 : 4000), anti-mouse (1 : 4000), and anti-goat (1 : 1000)) secondary detection antibodies were incubated for 2 h. Bio-Rad apparatus (Bio-Rad Laboratories, Richmond, CA) and Quantity One software version 4.6.2 (Bio-Rad Laboratories) were used to scan immune blots and for analysis.

### 2.11. Electrophoretic Mobility Shift Assay (EMSA)

Nuclear extracts were prepared as previously described. The NF-*κ*B double-stranded oligonucleotide corresponding to the NF-*κ*B consensus sequence (5′-AGTTGAGGGGACTTTCCCAGGC-3′) was obtained from Santa Cruz and was end-labeled with Terminal Deoxynucleotidyl Transferase (TdT) using Biotin-11-dUTP (Pierce). Nuclear extracts (8 *μ*g) were incubated at room temperature for 20 min with 1 *μ*L EMSA/Gel-Shift buffer. For competition studies, samples were also incubated with either 100-fold excess of unlabeled (cold) oligonucleotide or unlabeled mutant oligonucleotide. DNA-protein complexes were resolved on a 5% nondenaturing polyacrylamide gel with 20 mA for 3 h in 0.5× Tris-Borate EDTA. After UV cross linking, samples were blocked and incubated with streptavidin-HRP conjugate (15 mL). Hybridized samples were developed and analyzed using a Hybridization Incubator (WD-9403E, Beijing).

### 2.12. Enzyme-Linked Immunosorbent Assay (ELISA)

The levels of IL-1*β* and IL-13 in cytosolic brain fractions and in serum were analyzed by enzyme-linked immunosorbent assay (Bender, no. 81-BMS630/96T; Abcam, no. ab100766/96T).

## 3. Statistical Analysis

The software SPSS 16.0 for Windows (SPSS Inc, Chicago, IL) was used to conduct statistical analysis. All values are expressed as mean ± SD (standard deviation). Data were analyzed by a one-way ANOVA, and intergroup differences were detected by multivariate analysis, followed by LSD tests. The neurological deficit scores were expressed as median (range) and were analyzed with Kruskal-Wallis test, followed by the Mann-Whitney *U *test with Bonferroni correction. Values of *P* < 0.05 were considered as statistically significant.

## 4. Results

### 4.1. EA and NBD Peptide Treatment Significantly Declined the Ischemia/Reperfusion Damage after Focal Cerebral Ischemia/Reperfusion

#### 4.1.1. Neurobehavioral Evaluation

**Figure 1:**
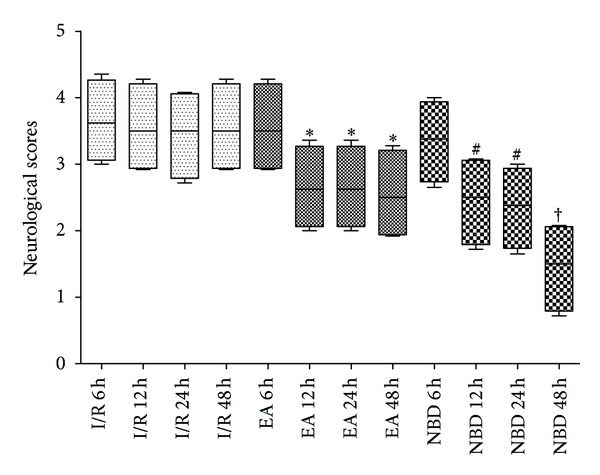
Neurological behavior scores from 6 h to 48 h after reperfusion in the rats with 2 h of MCAO. The neurological behavior scores in I/R group were worse from 6 h to 48 h. The treatment with EA and NBD peptide significantly improved the neurological scores, *P < 0.05, ^#^ *P* < 0.05 versus I/R group. There was more significant improvement in NBD peptide group than in EA group, ^†^ *P* < 0.05.

After focal cerebral I/R, the rats showed neurological deficit behavior at every time point (Figure 1). From 6 h to 48 h after reperfusion, compared with the I/R group, the neurobehavioral scores of EA group and NBD group were improved (*P* < 0.05) (Figure 1). The effect in NBD group was more remarkable than that in EA group (*P* < 0.05) (Figure 1). These results suggested that EA treatment and NBD treatment both ameliorated the neurologic deficit symptoms caused by I/R and promoted the movement recovery degree. It showed that EA could alleviate the ischemic lesion in the super early stage of focal cerebral I/R.

#### 4.1.2. The Infarct Volumes Value after MCAO/R

**Figure 2:**
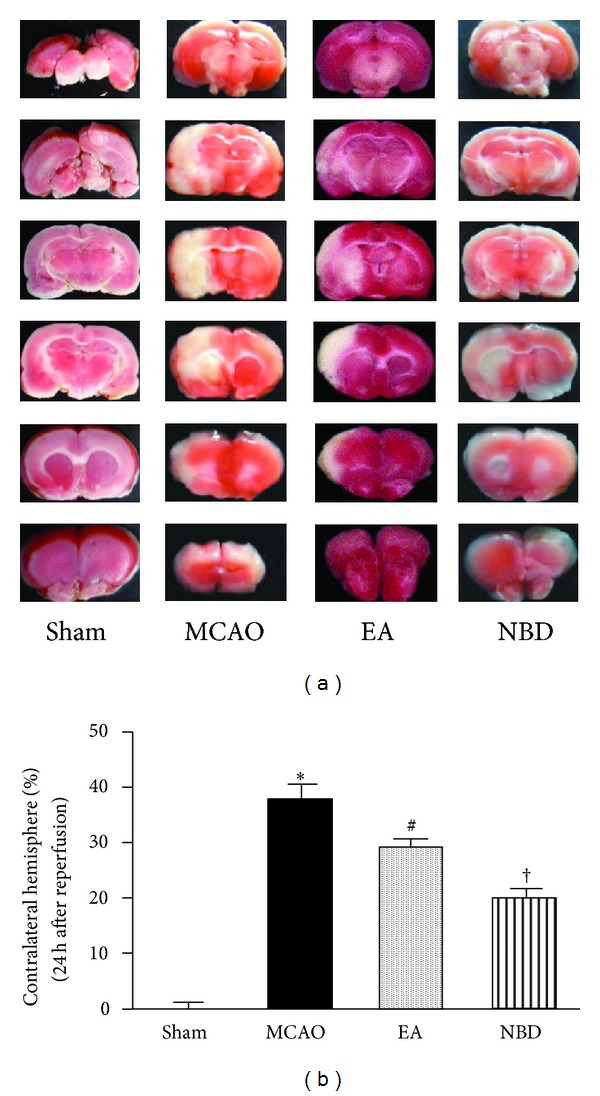
Infarct sizes at 24 h after reperfusion in the rats with 2 h of MCAO (5 rats in each group, total 20 rats). (a) Representative 2,3,5-triphenyltetrazolium chloride staining of the cerebral infarct in comparable sections of rat brain from 4 groups at 24 h after reperfusion. (b) Quantification of infarct volume at 24 h after reperfusion. The EA and NBD peptide treatment significantly reduced the infarct volume, *P < 0.05, ^#^ *P* < 0.05 versus I/R group. And the effect of NBD peptide was remarkable than EA group, ^†^ *P* < 0.05.

The infarct volume in EA group showed a smaller brain infarct volume compared with the I/R group (*P* < 0.05) (Figure 2). The brain infarct volume of NBD group was much more significantly reduced than that in EA group and I/R group (*P* < 0.05) (Figure 2). The data suggested EA and NBD peptide treatment could remarkably reduce the infarct size. Moreover, the effect of NBD peptide is more significant than EA treatment.

### 4.2. After EA and NBD Peptide Treatment, the Level of IL-1*β* Was Decreased and the Level of IL-13 Was Increased in Focal Cerebral Ischemia/Reperfusion Rats

**Figure 3:**
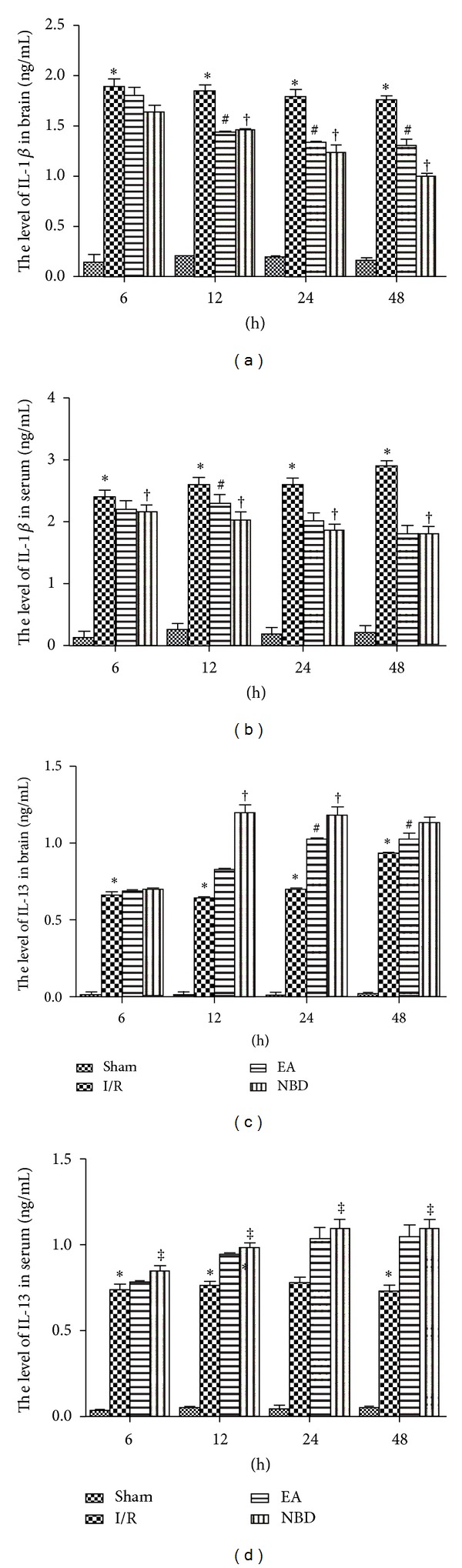
Levels of IL-1*β* and IL-13 in focal ischemic brain tissue and serum of rats among sham, I/R group, EA group, and NBD peptide group at different timepoints (5 rats in each group per time point, total 80 rats). (a) IL-1*β* levels in the brain of the I/R group increased significantly from 6 h to 48 h which were higher than those in EA group and NBD group, *P < 0.05. IL-1*β* levels in the EA group and NBD peptide group decreased gradually, ^#^ *P* < 0.05, ^†^ *P* < 0.05. (b) In serum, IL-1*β* levels peaked at 48 h in the I/R group and was significantly higher than those in the EA group and NBD group, *P < 0.05. ^†^ *P* < 0.05. IL-1*β* levels in the EA group decreased gradually even at 12 h, ^#^ *P* < 0.05. (c) In the brain, IL-13 peaked in the I/R group at 48 h, although these levels were lower than those in the EA group and NBD group, *P < 0.05. IL-13 levels peaked in the EA group at 24 h and in the NBD group at 12 h, and 24 h were significantly higher, ^#^ *P* < 0.05, ^†^ *P* < 0.05 versus I/R group. (d) There were no significant changes in the serum IL-13 levels in the I/R group at any of the timepoints, and these levels were significantly lower than those in the EA group and NBD group, *P < 0.05. The levels in NBD group were higher, ^‡^ *P* < 0.05 versus EA group^‡^.

After focal cerebral I/R, the levels of IL-1*β* increased significantly from 6 h to 48 h both in the brain (Figures 3(a) and 3(b)) and in serum (Figures 3(c) and 3(d)). However, in EA group and NBD group, IL-1*β* levels decreased significantly and were lower than that in I/R group (*P* < 0.05) (Figures 3(a) and 3(b)). The IL-13 levels were increased in the I/R group, especially at 48 h in brain and at 24 h in serum. In the EA group and NBD group in the brain and serum, the IL-13 levels were both increased which were significantly higher than those in I/R group at the same time points (*P* < 0.05) (Figures 3(c) and 3(d)). The IL-13 levels in the EA group peaked at 24 h in the brain and at 48 h in serum and peaked at 12 h and 24 h in NBD group (Figures 3(c) and 3(d)). These data suggested that EA and NBD peptide treatment effectively reduced IL-1*β* levels and increased IL-13 levels in brain and serum. Therefore, the EA and NBD peptide treatment effectively reduce the quantity of IL-1*β* and restrain the damage caused by inflammatory factor during the early stage. But the peak time points of NBD peptide and EA treatment are not the same.

### 4.3. EA and NBD Peptide Treatment Probably Inhibits NF-*κ*B Signaling Pathway Activation through Regulating the NF-*κ*B Nuclear Translocation

**Figure 4:**
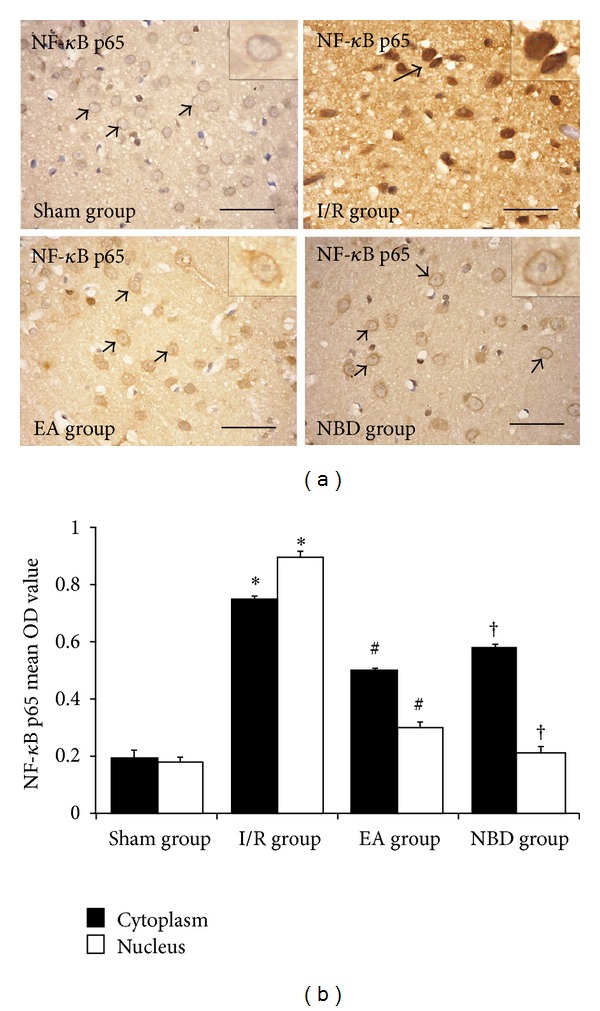
Immunohistochemical analysis of the nuclear translocation of NF-*κ*B p65 in the sham, I/R group, EA group, and NBD group (5 rats in each group, total 20 rats). In the temporal neocortex of sham group, the immunoreactive staining occurred less in the cytoplasm and nucleus. In the I/R group, strong immunoreactive staining occurred in the cytoplasm and nucleus, especially in the nucleus, *P < 0.05. In the EA group, immunoreactive staining was predominantly detected in the cytoplasm rather than in the nucleus, ^#^ *P* < 0.05. In the NBD group, immunoreactive staining was also maintained in the cytoplasm, ^†^ *P* < 0.05. Comparison of the mean OD value showed that NF-*κ*B p65 was mainly expressed in the nucleus after focal ischemia/reperfusion, and expression of NF-*κ*B p65 protein in the nucleus in the EA group and NBD group was significantly reduced, ^#^ *P* < 0.05, ^†^ *P* < 0.05.

Immunohistochemical analysis of NF-*κ*B p65 protein expression was conducted after focal cerebral I/R. In sham group, the NF-*κ*B p65 protein was predominantly expressed in the cytoplasm (Figures 4(a) and 4(b)). However, after focal cerebral I/R, expression was observed both in the cytoplasm and nucleus of the neurons in the ischemic penumbra area, although higher expression of NF-*κ*B p65 protein was detected in the nucleus than that in the cytoplasm (*P* < 0.05) (Figures 4(a) and 4(b)). Significant changes of NF-*κ*B p65 protein expression were observed in the EA group and NBD group, which were predominantly detected in the cytoplasm rather than in the nucleus (*P* < 0.05) (Figures 4(a) and 4(b)). These observations demonstrated that the differential localization of the NF-*κ*B p65 protein in the cytoplasm and nucleus was altered after EA and NBD peptide treatment, thus indicating that EA and NBD peptide may effectively inhibit the process of NF-*κ*B nuclear translocation.

### 4.4. EA and NBD Peptide Treatment Regulated the NF-*κ*B p65/I*κ*B*α* Feedback Loop to Inhibit the NF-*κ*B Nuclear Translocation

**Figure 5:**
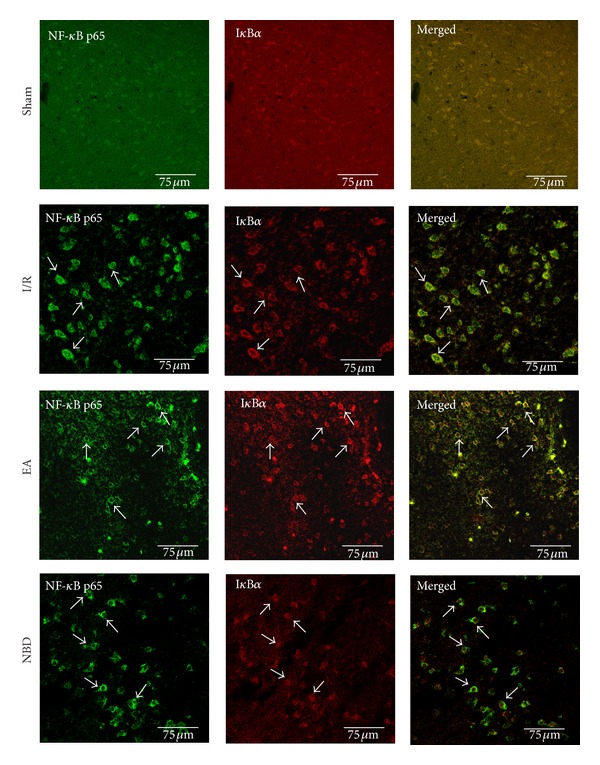
Double-immunofluorescent labeling the NF-*κ*B p65 (green) and I*κ*B*α* (red) in the cells in the ischemic cortex (5 rats in each group, total 20 rats). It showed that NF-*κ*B p65 and I*κ*B*α* were both poorly expressed in the sham group. In the I/R group, the NF-*κ*B p65 protein was expressed in cytoplasm and nucleus, especially highly in the nucleus; I*κ*B*α* protein was poorly expressed in cytoplasm and nucleus. In the EA group and NBD group, the NF-*κ*B p65 protein was mainly expressed in the cytoplasm than in the nucleus, and the I*κ*B*α* protein was expressed highly in the cytoplasm and nucleus.

The protein expression and localization of NF-*κ*B p65 (green) and I*κ*B*α* (red) in the ischemic penumbra area were examined by double-immunofluorescence staining. As shown in Figure 5, in sham group, low levels of NF-*κ*B p65 and I*κ*B*α* protein expression were observed throughout the hemisphere. After focal cerebral I/R, NF-*κ*B p65 protein expression was observed both in the nucleus and cytoplasm, meanwhile there was a little expression of I*κ*B*α* protein in the nucleus (Figure 5). In contrast, NF-*κ*B p65 protein expression was remarkably reduced in the nucleus in EA group and NBD group; the I*κ*B*α* protein expression in the cytoplasm and nucleus was significantly increased (Figure 5). Double-immunofluorescence analysis of NF-*κ*B p65 and I*κ*B*α* protein expression demonstrated that NF-*κ*B p65 protein was mainly localized in the cytoplasm, while I*κ*B*α* was highly expressed in nucleus and cytoplasm after EA and NBD peptide treatment.

**Figure 6:**
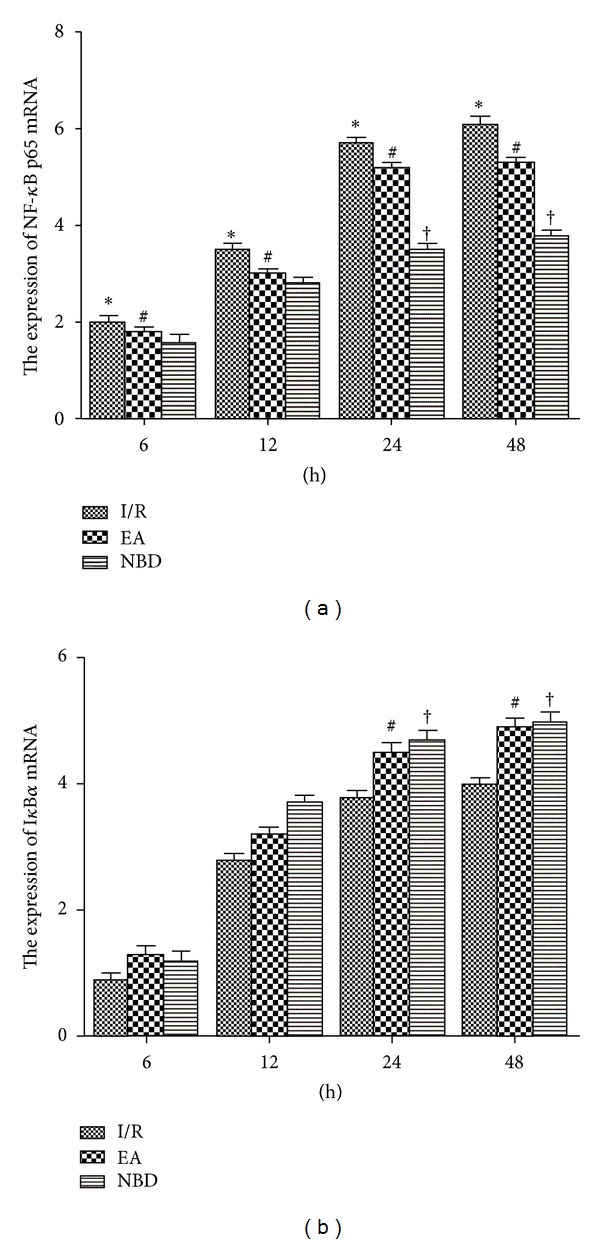
Fluorescent Quantitative-PCR analysis of the NF-*κ*B p65 and I*κ*B*α* mRNA expression of the sham group, I/R group, EA group, and NBD group in the ischemic brain tissue (5 rats in each group per time point, total 80 rats). (a) The expression of NF-*κ*B p65 mRNA was significantly increased in the I/R group compared with that in the EA and NBD groups, *P < 0.05. At 24 h and 48 h, the NF-*κ*B p65 mRNA in the EA group was higher, ^#^ *P* < 0.05, ^†^ *P* < 0.05 versus NBD group. (b) The I*κ*B*α* mRNA expression in EA group and NBD group was higher, ^#^ *P* < 0.05, ^†^ *P* < 0.05 versus I/R group.

The fluorescent quantitation-PCR analysis showed that the expression of NF-*κ*B p65 mRNA in I/R group was significantly increased at 24 h and 48 h which were higher than those in EA group and NBD group (*P* < 0.05) (Figure 6(a)). Expression of I*κ*B*α* mRNA in the EA group and NBD group was obviously increased and was higher than that in I/R group (*P* < 0.05) (Figure 6(b)).

**Figure 7:**
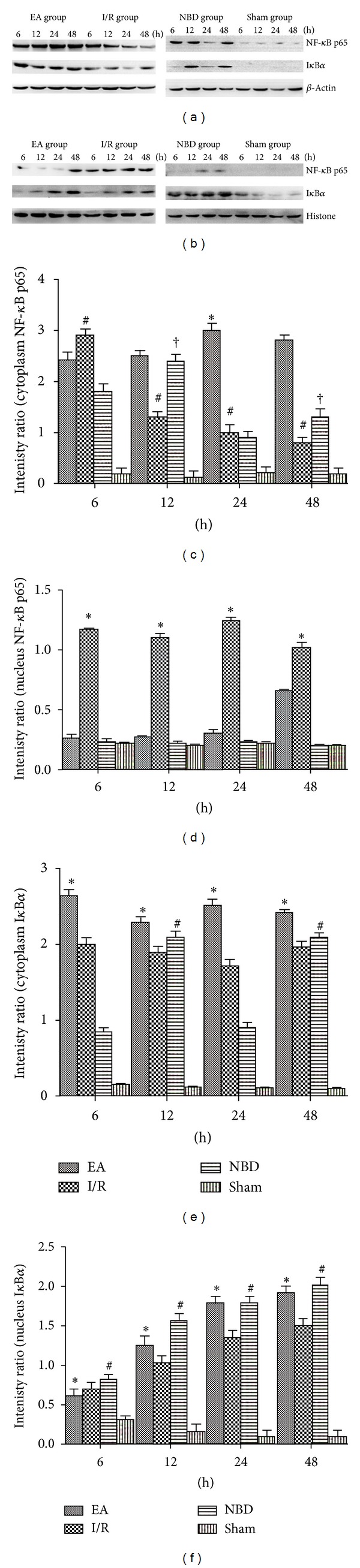
Western blot analysis of the NF-*κ*B p65 and I*κ*B*α* protein expression in the sham group, I/R group, EA group, and NBD group in ischemic brain tissue (5 rats in each group per time point, total 80 rats). (a)-(b) Representative Western blot images showing bands of NF-*κ*B p65 and I*κ*B*α* proteins in the cytoplasm and nucleus at 6 h, 12 h, 24 h, and 48 h. (c)–(f) Comparison of the mean intensity ratio of immunoblotting in these groups at each timepoint. (a), (c), (e) The NF-*κ*B p65 and I*κ*B*α* protein expression in cytoplasm. (b), (d), (f) The NF-*κ*B p65 and I*κ*B*α* protein expression in the nucleus.

The expression of NF-*κ*B p65 and I*κ*B*α* proteins were analysed by Western blot among the sham group, I/R group, EA group, and NBD group. In the I/R group, the expression of NF-*κ*B p65 protein in the cytoplasm decreased gradually, meanwhile low levels of I*κ*B*α* protein were detected. Compared with the I/R group, the expression of NF-*κ*B p65 and I*κ*B*α* protein in the EA group and NBD group was increased (*P* < 0.05) (Figures 7(a), 7(c), and 7(e)). In contrast in the nucleus, the expression of NF-*κ*B p65 protein was significantly increased in the I/R group and obviously decreased in the EA group and NBD group (*P* < 0.05) (Figures 7(b) and 7(d)). The expression of I*κ*B*α* protein was obviously increased in the EA group and NBD group, which were higher than that in the I/R group (*P* < 0.05) (Figures 7(b) and 7(f)). These data showed that EA and NBD peptide treatment effectively altered the expression of NF-*κ*B p65 and I*κ*B*α* proteins in both cytoplasm and nucleus after focal cerebral I/R. They decreased NF-*κ*B p65 protein expression in the nucleus and maintained it in the cytoplasm, while I*κ*B*α* protein expression was increased in the nucleus and cytoplasm. Therefore, these data indicated that the mechanism by which EA and NBD peptide treatment regulated NF-*κ*B nuclear translocation was related to the high expression of I*κ*B*α* in the cytoplasm and nucleus.

### 4.5. EA and NBD Peptide Treatment Regulated the Expression of IKK*α* and IKK*β* mRNA and Protein after Focal Cerebral Ischemia/Reperfusion

**Figure 8:**
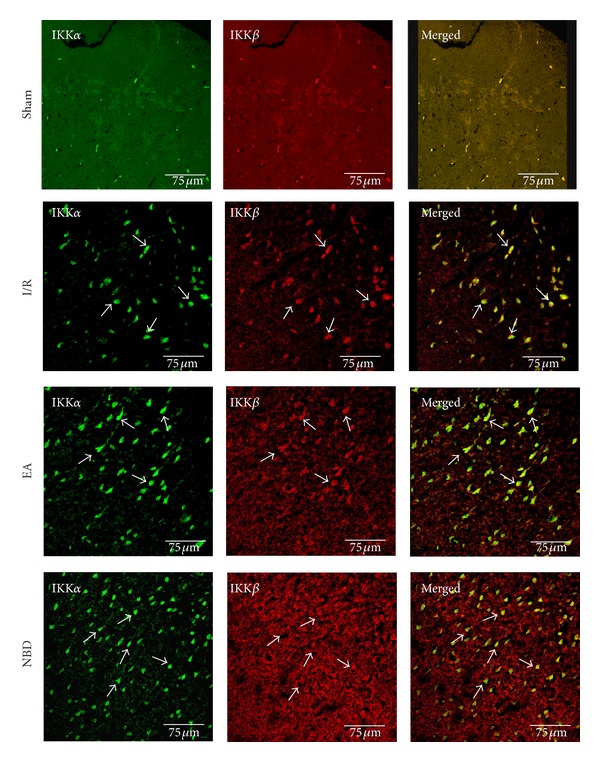
Double-immunofluorescent labeling the IKK*α* (green) and IKK*β* (red) protein in sham group, I/R group, EA group, and NBD group (with the same samples from Figure 5). It showed that IKK*α* and IKK*β* were strongly expressed in cytoplasm in the I/R group. The expression of IKK*β* was lowly expressed in EA group and NBD group compared with the I/R group, especially in the NBD group.

The expression of IKK*α* (green) and IKK*β* (red) protein was examined by double-immunofluorescence staining in the sham group, I/R group, EA group, and NBD peptide group. As shown in Figure 8, little expression of IKK*α* and IKK*β* proteins was observed in the sham group (Figure 8), and they were increased significantly after focal cerebral I/R (Figure 8) but were obviously decreased in the EA group and NBD group, especially decreased the IKK*β* expression (Figure 8).

**Figure 9:**
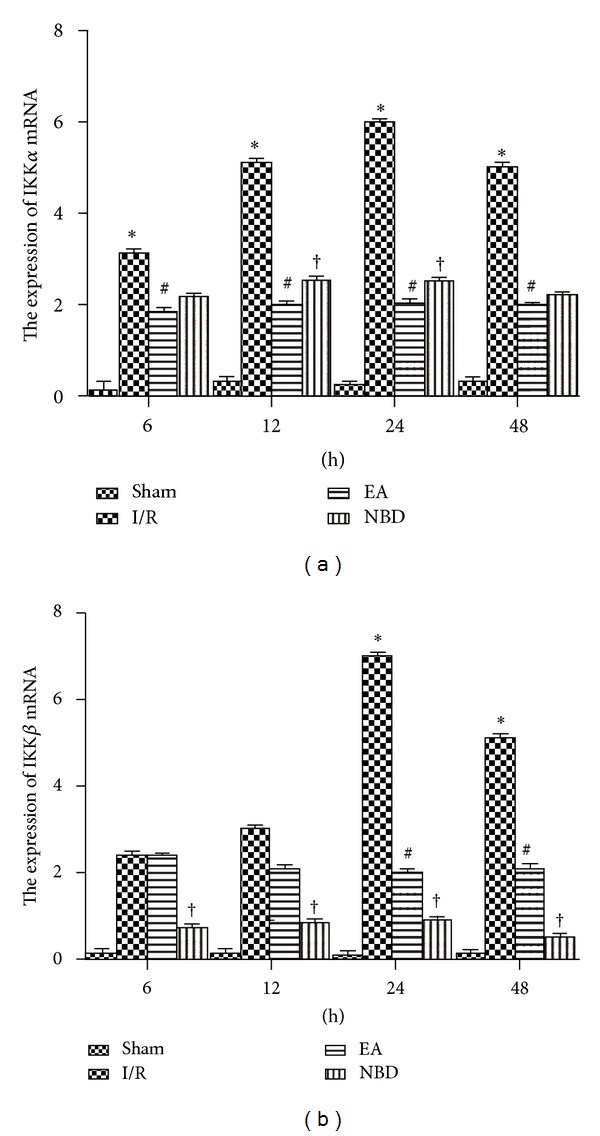
Fluorescent Quantitative-PCR analysis of the IKK*α* and IKK*β* mRNA expression of the sham group, I/R group, EA group, and NBD group in the ischemic brain tissue (with the same samples from Figure 6). (a) The expression of IKK*α* mRNA was significantly increased in the I/R group compared with that in the EA and NBD group, *P < 0.05. The IKK*α* mRNA expressed lowly in EA group, ^#^ *P* < 0.05 versus I/R group and NBD group. (b) The IKK*β* mRNA expression in I/R group was high at 24 h and 48 h, *P < 0.05 versus EA group and NBD group. It was lower in NBD group from 6 h to 48 h, ^†^ *P* < 0.05 versus EA group.

The fluorescent quantitation-PCR (Q-PCR) analysis showed that the expression of IKK*α* and IKK*β* mRNA in the I/R group was significantly increased (*P* < 0.05) (Figures 9(a) and 9(b)). The IKK*α* mRNA expression in the EA group was remarkably reduced, which was lower than that in I/R group (*P* < 0.05) (Figure 9(a)). The peak expression in the NBD group was higher than that in the EA group (*P* < 0.05) (Figure 9(a)). Compared with the I/R group, the expression of IKK*β* mRNA in the EA group was obviously decreased (*P* < 0.05) (Figure 9(b)). In the NBD group, IKK*β* mRNA expression was negligible from 6 h to 48 h (Figure 9(b)).

**Figure 10:**
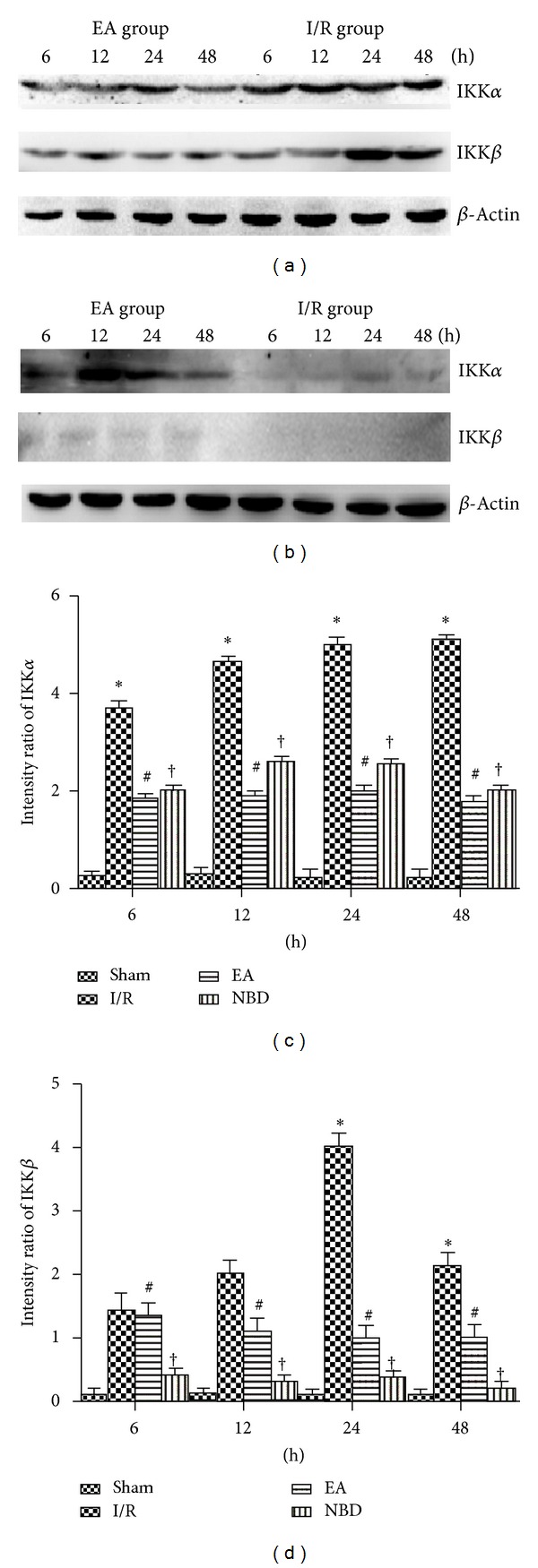
Western blot analysis of the IKK*α* and IKK*β* protein expression in the sham group, I/R group, EA group, and NBD group in ischemic brain tissue (with the same samples from Figure 7). (a)-(b) Representative Western blot images showing bands of IKK*α* and IKK*β* proteins at 6 h, 12 h, 24 h, and 48 h. Comparison of the mean intensity ratio of immunoblotting in these groups at each timepoint. (a), (b), (c) The IKK*α* protein in I/R group was highly expressed, *P < 0.05 versus EA and NBD group. The expression in EA group was lower, ^#^ *P* < 0.05 versus NBD group. (a), (b), (d) The IKK*β* protein was highly expressed at 24 h and 48 h, *P < 0.05 versus EA and NBD group. The IKK*β* protein scarcely expressed in NBD group, ^†^ *P* < 0.05 versus EA group.

The protein expression of IKK*α* and IKK*β* was analysed by Western blot. In the I/R group, IKK*α* protein expression was persistently increased from 6 h to 48 h (Figures 10(a) and 10(c)). The expression of IKK*α* protein was decreased in the EA group, which was lower than that in the I/R group (*P* < 0.05) (Figures 10(a) and 10(c)). In the NBD group, expression was also reduced but was still much higher than that in the EA group, especially at 12 h (*P* < 0.05) (Figures 10(b) and 10(d)). Expression of IKK*β* protein in the I/R group was significantly increased, especially at 24 h and 48 h (*P* < 0.05) (Figures 10(a) and 10(d)). Compared with the I/R group, expression was remarkably decreased in the EA group (*P* < 0.05) (Figures 10(a) and 10(d)). Furthermore, in the NBD group, IKK*β* protein expression was persistently low from 6 h to 48 h (*P* < 0.01) (Figures 10(b) and 10(d)). These data demonstrated that EA and NBD peptide treatment could effectively reduce the high levels of IKK*β* expression after focal cerebral I/R, especially at 24 h and 48 h. Compared with the NBD peptide group, EA treatment could else reduce IKK*α* protein expression.

### 4.6. EA and NBD Peptide Treatment Effectively Regulated the DNA Binding Capacity of NF-*κ*B

**Figure 11:**
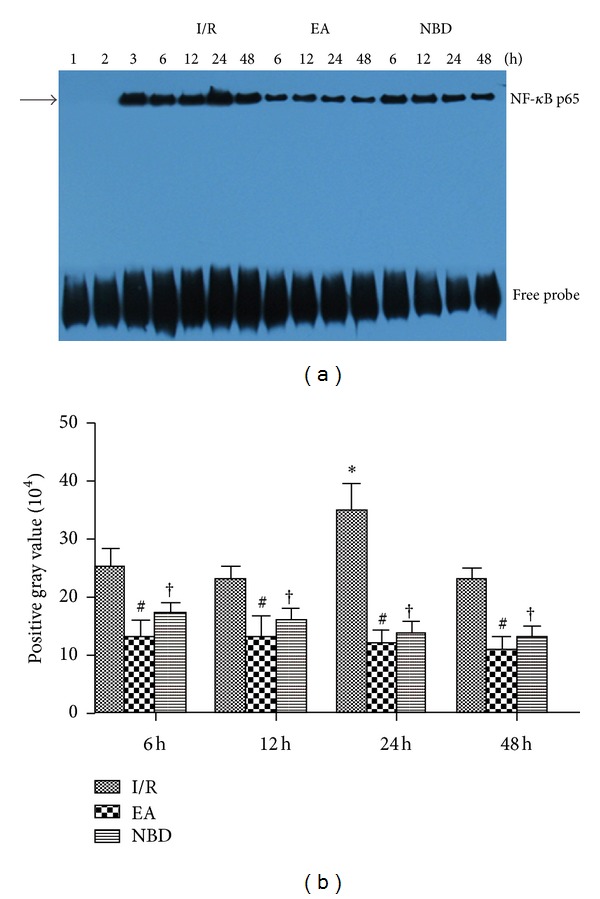
Electrophoretic mobility shift assay detection of NF-*κ*B activity in the I/R, EA, and NBD groups in focal ischemia/reperfusion cortex in rats (5 rats in each group per time point, total 80 rats). (a) lane 1: negative probe, lane 2: cold competition probe, lane 3: positive probe. (b) The highest activity of NF-*κ*B was at 24 h in the I/R group, which was significantly higher than that in the EA group and NBD group, *P < 0.05. The NF-*κ*B activity in EA group and NBD group shows no remarkable differences, ^#^ *P* > 0.05.

Electrophoretic mobility shift assays (EMSA) were used to further investigate theactivity of NF-*κ*B after focal cerebral I/R and the change in activity mediated by EA treatment and NBD peptide intervention. The activity of NF-*κ*B was increased after I/R, especially at 24 h and 48 h, indicating that the DNA binding capacity of NF-*κ*B was high in the I/R group (*P* < 0.05) (Figures 11(a) and 11(b)). In the EA group and NBD group, the activity of NF-*κ*B was reduced significantly (*P* < 0.05), and there were no remarkable differences between those (*P* > 0.05) (Figures 11(a) and 11(b)). The results suggest that EA and NBD peptide treatment effectively reduces the activity of NF-*κ*B.

## 5. Discussion

In the present study, we investigated the anti-inflammation effect and probable mechanism of EA on the NF-*κ*B signaling pathway after focal cerebral ischemia/reperfusion. First of all, we found that EA treatment effectively reduced the cerebral infarct volume and improved the neurobehavioral scores, it seemed able to alleviate the ischemic damage. Secondly, the EA treatment also regulated the levels of interleukin (IL-1*β* and IL-13) in brain and serum after focal cerebral I/R. More importantly than all of that, we observed that EA treatment reduced the expression of IKK*α* and IKK*β* protein, perhaps decline the function of IKK*α* and IKK*β*, and then regulated the NF-*κ*B p65/I*κ*B*α* feedback loop for the activation of NF-*κ*B signaling pathway effectively during very early stage of focal cerebral I/R, which demonstrated that EA has obviously protective and anti-inflammatory effect during the focal cerebral I/R injury, via a mechanism related to the suppression of the activation of the NF-*κ*B signaling pathway at a very early phase. Moreover, the effects on the IKK*α* and IKK*β* were remarkably different between EA treatment and NBD peptide.

The inflammatory reaction is an important pathological step in the process of focal cerebral I/R, which causes rapid neuronal death and aggravates the ischemic injury and body burden [30]. Therefore, effectively restraining the occurrence and development of the inflammation is considered to be an important target and direction of the treatment for focal cerebral I/R. The cytokines that cause the inflammatory response include TNF-*α*, IL-1*β*, cox-2, iNOS, and many other types of factors [31]. IL-1*β* is a nerve toxin and a major stimulus factor of the inflammatory reaction. Reducing the level of IL-1*β* can significantly ameliorate the ischemia infarction volume and the occurrence of inflammation [32]. On the contrary, the cytokines such as IL-10 and IL-13 exert better anti-inflammatory effect. Inflammatory reaction induced by IL-1*β* stimulation is significantly inhibited by IL-13 [33]. Focused on the inflammatory cytokines in the ischemic brain and serum, we observed that the level and the timing of the peak expression of IL-1*β* and IL-13 were regulated by EA and NBD peptide treatment. All of these data demonstrated that EA treatment could exert remarkable anti-inflammatory effect during the early stage of focal cerebral I/R which resembles the function of NBD peptide. However, the effect of NBD peptide is more remarkable on the regulation of cytokines.

In order to regulate the inflammatory injury occurred during the early stages after reperfusion, it is necessary to inhibit the activation of NF-*κ*B signaling pathway which is induced by focal cerebral I/R. During the activation process of NF-*κ*B signaling pathway, the interaction of p65 and I*κ*B*α* on nuclear translocation is the key step [34, 35]. In the present study, we observed that NF-*κ*B p65 expressed both in cytoplasm and nucleus, the NF-*κ*B p65 predominantly expressed in nucleus after focal cerebral I/R. It showed that the ischemia and anoxic pathological factors in the brain stimulated the p65 mainly expressed in the nucleus and promoted the NF-*κ*B nuclear translocation process. We observed that EA and NBD peptide treatment mainly maintained high cytosolic level of p65 and significantly reduced it in nucleus. The discovery demonstrated that EA effectively inhibited p65 nuclear translocation and maintained it in the cytoplasm, which we also found after NBD peptide treatment. According to the general understanding of the NF-*κ*B signal pathway, nuclear NF-*κ*B drives I*κ*B*α* expression generating a negative feedback loop [36, 37]. In the present study, we found that EA treatment significantly increased I*κ*B*α* expression in the cytoplasm and nucleus, especially expressed in the nucleus, and altered the NF-*κ*B p65 and I*κ*B*α* protein expression in nucleus. The results showed that EA treatment could regulate NF-*κ*B/I*κ*B*α* feedback loop in the nucleus and cytoplasm. Above all, it reveals that EA promotes the inactive heterotrimer generated in nucleus through increasing I*κ*B*α* expression in the NF-*κ*B/I*κ*B*α* feedback loop and then maintains the high cytosolic level of p65. It can be speculated that inhibition of NF-*κ*B nuclear translocation is likely to be one of the most important mechanisms by which EA inhibits NF-*κ*B activation.

The function of I*κ*B*α* is regulated by the I*κ*B kinases (IKKs) in the NF-*κ*B signal pathway, and NF-*κ*B activation depends on the IKK phosphorylation process [38]. Both IKK*α* and IKK*β* phosphorylate I*κ*B*α* at Ser 32 and Ser 36, although IKK*α* is less efficient and consequently cannot complement IKK*β* knockout cells [18, 38]. In this study, we observed that the expression of IKK*α* and IKK*β* was significantly increased after focal cerebral I/R, which demonstrated that the massive expression of IKK*α* and IKK*β* could be required for NF-*κ*B activation. However, EA treatment remarkably reduced the expression of IKK*α* and IKK*β*, especially for the IKK*β*. The results of the EA group were obviously different from the NBD peptide effect which focuses solely on the decreased expression of IKK*β* rather than IKK*α*. The results of the NBD peptide group were consistent with the characteristics of NBD peptide. Thus, we have demonstrated the effect of EA on the IKK*α* and IKK*β* for the first time in the focal cerebral I/R rats.

Furthermore, the different effect and mechanism between EA treatment and NBD peptide on IKK*α* and IKK*β* were also prompted. 

Besides, the key step in this process is nuclear translocation; binding of the p65/p50 dimers to the *κ*B sequence on DNA is another important step in the activation of NF-*κ*B signaling pathway [39]. We found that the DNA binding ability of NF-*κ*B was increased after focal cerebral ischemia/reperfusion and was significantly reduced by EA treatment and NBD peptide intervention. This demonstrates that EA treatment and NBD peptide inhibit the NF-*κ*B signaling pathway through inhibiting the ability of NF-*κ*B binding DNA.

In summary, EA effectively inhibits the activation of NF-*κ*B signaling pathway perhaps through reducing the expression of IKK*α* and IKK*β*, which is highly expressed after focal cerebral ischemia/reperfusion. The function of IKK*α* and IKK*β* in the NF-*κ*B pathway maybe inhibited, which probably attenuates I*κ*B*α* phosphorylation, resulting in increased I*κ*B*α* expression in the NF-*κ*B/I*κ*B*α* feedback loop in the nucleus, which may block the nuclear translocation of NF-*κ*B. Furthermore, EA reduced the DNA binding ability of NF-*κ*B and thus effectively reduced activation of the NF-*κ*B pathway. Compared with the NBD peptide, EA treatment mediated a reduction in the expression of IKK*α*. It can be speculated that the differences in these observations reflect the different mechanism of EA treatment and NBD peptide. Unfortunately, we have concentrated only on the canonical pathway. Moreover, because the effect of IKK*α* serves an anti-inflammatory function by phosphorylating Rel A and c-Rel at sites that accelerate their nuclear turnover [40], the effect and mechanism of EA on IKK*α* in the noncanonical pathway and the phosphorylation process of IKKs will be investigated in future studies. And the effect of EA on other signaling pathways during the inflammation also is considered to be our next research targets.

In short, we infer that inhibition of the NF-*κ*B signaling pathway activation is involved in the mechanism by which EA treatment ameliorates the inflammatory injury occurred during the early stages of focal cerebral ischemia/reperfusion.

## 6. Conclusions

In conclusion, the mechanism by which EA inhibits activation of the NF-*κ*B signaling pathway and declines the inflammatory injury involves multiple targets and links. EA treatment could reduce the expression of IKK*α* and IKK*β* and may restrain the function of those to regulate the activation of the NF-*κ*B signaling pathway and ameliorate the inflammation in the early stages of focal cerebral ischemia/reperfusion.

## Figures and Tables

**Table 1:** Primer sequences and annealing temperatures employed for Q-PCR.

Primer name	Sequence	Annealing temperature
NF-*κ*B p65	F 5′-ATCGTGGAGCACTTGGTGACT-3′ R 5′-GCCCTGGTAGGTTACTCTGTTGA-3′	55.2°C
I*κ*B*α*	F 5′-GTTTCCCCTCATCTTTCCCTCA-3′ R 5′-GGGTGCGTCTTAGTGGTATCTGT-3′	56.7°C
IKK*α*	F 5′-GTCAGGAGAAGTTCGGTTTGA-3′ R 5′-ATTCCAGTTTCACGCTCATGGAT-3	54.2°C
IKK*β*	F 5′-GTCTTGTTGATGGTTCCTGACT-3′ R 5′-AAGACAAACGAGGGCCTCACAT-3′	56.0°C
GAPDH	F 5′-CTTAGCACCCCTGGCCAAG-3′ R 5′-GATGTTCTGGAGAGCCCCG-3′	54.0°C

## Abbreviations

EA: Electroacupuncture
NF-*κ*B: Nuclear factor-*κ*appaB
NBD peptide: NEMO Binding Domain peptide
I/R: Ischemia/reperfusion
SD: Sprague-Dawley
PFA: Paraformaldehyde
PBS: Phosphate buffer saline
PVDF: Polyvinylidene fluoride
EMSA: Electrophoretic mobility shift assay
RHD: Rel-homology domain
NES: Nuclear export sequences.
